# Author Correction: Simulating a chemically fueled molecular motor with nonequilibrium molecular dynamics

**DOI:** 10.1038/s41467-023-35980-9

**Published:** 2023-01-24

**Authors:** Alex Albaugh, Todd R. Gingrich

**Affiliations:** Department of Chemistry, Northwestern University, 2145 Sheridan Road, Evanston, IL 60208 USA

**Keywords:** Thermodynamics, Statistical mechanics, Computational nanotechnology

Correction to: *Nature Communications* 10.1038/s41467-022-29393-3, published online 22 April 2022

The original version of this Article contained an error in Equation (12) of the main manuscript and in Equation (C1) of its associated Supplementary Information.

In Equation (12) of the main manuscript, the maximum extension between the particle pair, *r*_max,*ij*_, appearing before the logarithm, was incorrectly given as the displacement vector, **r**_*ij*_. The equation incorrectly read:

$${U}_{{{{\rm{FENE}}}}}({{{{\bf{r}}}}}_{ij})=-\frac{1}{2}{k}_{{{{\rm{F}}}},ij}{{{{\bf{r}}}}}_{ij}^{2}\,\log \left[1-{\left(\frac{|{{{{\bf{r}}}}}_{ij}|}{{r}_{\max,ij}}\right)}^{2}\right]. \; (12)$$

The correct form of Equation (12) is:

$${U}_{{{{\rm{FENE}}}}}({{{{\bf{r}}}}}_{ij})=-\frac{1}{2}{k}_{{{{\rm{F}}}},ij}{r}_{\max,ij}^{2}\,\log \left[1-{\left(\frac{|{{{{\bf{r}}}}}_{ij}|}{{r}_{\max,ij}}\right)}^{2}\right]. \; (12)$$

Furthermore, in Equation (C1) of the Supplementary Information associated with this Article, the maximum extension, *r*_max,c_, before the logarithm, was incorrectly given as the vector between the central particle and the center of mass of the four tetrahedron particles, **r**_c_. The equation incorrectly read:

$${U}_{{{{\rm{FENE}}}}}({{{{\bf{r}}}}}_{c})=-\frac{1}{2}{k}_{{{{\rm{constraint}}}}}{{{{\bf{r}}}}}_{c}^{2}\,\log \left[1-{\left(\frac{|{{{{\bf{r}}}}}_{c}|}{{r}_{\max,{{{\rm{c}}}}}}\right)}^{2}\right],\; ({{{\rm{C}}}}1)$$

The correct form of Equation (C1) is:

$${U}_{{{{\rm{FENE}}}}}({{{{\bf{r}}}}}_{{{{\rm{c}}}}})=-\frac{1}{2}{k}_{{{{\rm{constraint}}}}}{r}_{\max,{{{\rm{c}}}}}^{2}\,\log \left[1-{\left(\frac{|{{{{\bf{r}}}}}_{{{{\rm{c}}}}}|}{{r}_{\max,{{{\rm{c}}}}}}\right)}^{2}\right], \; ({{{\rm{C}}}}1)$$

These have been corrected in both the PDF and HTML versions of the Article. The HTML has also been updated to include a corrected version of the Supplementary Information.

## Supplementary information

Updated Supplementary Information

