# Supplementary material for: Simulating a chemically fueled molecular motor with nonequilibrium molecular dynamics
Source: Nat Commun. 2022 Apr 22;13:2204. doi: 10.1038/s41467-022-29393-3 (PMC9033874; doi:10.1038/s41467-022-29393-3)
Supplement: Supplementary file 1 — Supplementary Information [file 41467_2022_29393_MOESM1_ESM.pdf]

Supplementary Information for "Simulating a Chemically-Fueled Molecular Motor with Nonequilibrium Molecular Dynamics"

Alex Albaugh[1] and Todd R. Gingrich[1,2]

[1] *Department of Chemistry, Northwestern University,*
2145 Sheridan Road, Evanston, Illinois 60208, USA
[2] *To whom correspondence should be addressed. E-mail:* `todd.gingrich@northwestern.edu`

Appendix A: Movie Files

Three SI Movies depict representative simulations. All movies show dynamics at moderate fuel concentration with $\mu'_{\mathrm{FTC}} = 0.5$, $\mu'_{\mathrm{ETC}} = -10$, and $\mu'_{\mathrm{C}} = -10$. The file `Motor_Operation.gif` shows the dynamics of the full simulation cell with frames taken every 50,000 time steps. The movie shows a clear preference for clockwise motion over counterclockwise motion. The `Cleave_Close.gif` and `Attach_Close.gif` movie files show representative pathways for the $k_{\mathrm{cleave,close}}$ and $k_{\mathrm{attach,close}}$ pathways, respectively. In these movies each frame represents 50 individual time steps. The cleave pathway shows that the transitions happens with a simple unbinding of a red C particle from the close catalytic site. The attach pathway shows a transition that occurs as an FTC cluster decomposes into ETC and C at the catalytic site, leaving behind a C particle as a blocking group. These two pathways are not time-reverses of each other.

In all movie files we translated and rotated the system configuration in each frame to give a clear view of the shuttling ring's relative motion in the frame of a stationary large ring. We first translated the motor's center of mass to the center of the frame and then performed rotations so that the (orange) binding sites were at, approximately, the 12 o'clock and 6 o'clock positions and the large ring was in a plane approximately perpendicular to the observer.

Appendix B: Grand Canonical Monte Carlo Details

To target the grand Canonical distribution of Eq. (15), GCMC trial moves are attempted throughout a volume V_0. Using the Metropolis criterion, the probability of inserting an additional i molecule and accepting this trial move is

$$P^{\mathrm{acc}}_{i\ \mathrm{addition}}(\mathbf{r},\mathbf{p}\to\mathbf{r}',\mathbf{p}') = \min\left[1, \frac{P(\mathbf{r}',\mathbf{p}')}{P(\mathbf{r},\mathbf{p})}\frac{P^{\mathrm{gen}}_{i\ \mathrm{removal}}(\mathbf{r}',\mathbf{p}'\to\mathbf{r},\mathbf{p})}{P^{\mathrm{gen}}_{i\ \mathrm{addition}}(\mathbf{r},\mathbf{p}\to\mathbf{r}',\mathbf{p}')}\right] \tag{B1}$$

where P^{gen} is the probability of generating such a trial move. Here we adopt a notation where $\mathbf{r},\mathbf{p}$ denotes the positions and momenta, respectively, of all particles in the initial system with $N_i(\mathbf{r})$ molecules of species i (where i can be FTC, ETC, or C). The trial move inserts an additional molecule of i and the positions and momenta of all particles in this configuration is given by $\mathbf{r}',\mathbf{p}'$. The probabilities $P(\mathbf{r},\mathbf{p})$ and $P(\mathbf{r}',\mathbf{p}')$ are given by the grand canonical distribution, Eq. (15).

The probability of generating the insertion move is the probability of uniformly selecting that the next move would be an insertion of species i times the probability that the inserted species will be given a particular label in the list of i species times the Boltzmann probability of the inserted molecular configuration:

$$P^{\mathrm{gen}}_{i\ \mathrm{insertion}}(\mathbf{r},\mathbf{p}\to\mathbf{r}',\mathbf{p}') = \frac{1}{6}\frac{1}{N_i(\mathbf{r}')}\frac{e^{-\beta H(\mathbf{r}^0,\mathbf{p}^0)}}{Z_i^0}. \tag{B2}$$

Here $\mathbf{r}^0$ and $\mathbf{p}^0$ are the positions and momenta, respectively, of only the inserted molecule's particles. The trial configuration $\mathbf{r}',\mathbf{p}'$ is then $\mathbf{r},\mathbf{p}\cup\mathbf{r}^0,\mathbf{p}^0$ The total energy $H(\mathbf{r}^0,\mathbf{p}^0)$ is the internal energy of the inserted molecule if it were in isolation, with a normalizing partition function Z_i^0 for the single species i in a volume equal to the volume available for GCMC moves, V_0:

$$Z_i^0 = \frac{1}{h^{3n_i}}\int \mathrm{d}\mathbf{p}^0 \int_{V_0} \mathrm{d}\mathbf{r}^0 e^{-\beta H(\mathbf{r}^0,\mathbf{p}^0)}. \tag{B3}$$

Here h is Planck's constant and n_i is the number of particles in species i. Practically, to achieve configurations to insert that come from this Boltzmann ensemble we first pre-sample the FTC and ETC species with standard Markov chain

Monte Carlo to build a library of configurations [1]. We then uniformly choose a configuration from this library, rotate that configuration into a uniformly sampled orientation, then place it inside the volume V_0 at a uniformly sampled location. Random velocities for all the inserted particles are sampled from the Boltzmann ensemble independently of the positions. The library generation and random rotation are not necessary for the C species since it is a single spherically symmetrical particle with no internal potential energy.

The probability of generating the reversal of the insertion move is simply the probability of choosing to remove a species i particle times the probability of selecting the particular one of the $N_i(\mathbf{r}')$ particles to delete:

$$P^{\text{gen}}_{i\ \text{removal}}(\mathbf{r}', \mathbf{p}' \to \mathbf{r}, \mathbf{p}) = \frac{1}{6}\frac{1}{N_i(\mathbf{r}')}. \tag{B4}$$

Substituting Eq. (15), Eq. (B2), and Eq. (B4) into Eq. (B1) yields

$$P^{\text{acc}}_{i\ \text{addition}}(\mathbf{r}, \mathbf{p} \to \mathbf{r}', \mathbf{p}') = \min\left[1, \frac{1}{N_i(\mathbf{r}')} e^{-\beta[H(\mathbf{r}',\mathbf{p}') - H(\mathbf{r},\mathbf{p}) - H(\mathbf{r}^0,\mathbf{p}^0)]} e^{\beta\mu_i} Z_i^0\right]. \tag{B5}$$

Recognizing that kinetic energy is purely additive across particles, $K(\mathbf{p}') = K(\mathbf{p}) + K(\mathbf{p}^0)$, and that $A_i^0 = -\log Z_i^0/\beta$ is the Helmholtz free energy of a single molecule of species i in volume V_0, we can simplify even further, yielding

$$P^{\text{acc}}_{i\ \text{addition}}(\mathbf{r}, \mathbf{p} \to \mathbf{r}', \mathbf{p}') = \min\left[1, \frac{1}{N_i(\mathbf{r}')} e^{-\beta[U(\mathbf{r}') - U(\mathbf{r}) - U(\mathbf{r}^0)]} e^{\beta\mu_i'}\right]. \tag{B6}$$

In a similar way, the acceptance probability of a trial move that removes a molecule of species i is given as

$$P^{\text{acc}}_{i\ \text{removal}}(\mathbf{r}, \mathbf{p} \to \mathbf{r}', \mathbf{p}') = \min\left[1, N_i(\mathbf{r}) e^{-\beta[U(\mathbf{r}') - U(\mathbf{r}) + U(\mathbf{r}^0)]} e^{-\beta\mu_i'}\right]. \tag{B7}$$

In both cases we introduce the definition $\mu_i' \equiv \mu_i - A_i^0$ as a modified chemical potential that is the true external chemical potential less the free energy of the corresponding species at a reference state with a single molecule in volume V_0 and inverse temperature β. We need not calculate this free energy and can instead use μ_i' as the "knob" to turn to adjust the concentration of species in the simulation box. We would only need to calculate A_i^0 if we wanted to know the true absolute external chemical potential μ_i. As μ_i' becomes larger the concentration of species in the simulation box will increase and as μ_i' decreases the concentration will decrease. By setting a high chemical potential for FTC and low chemical potential for ETC and C, we can set up a NESS as the FTC decomposes to form ETC and C.

To summarize, a GCMC step begins by uniformly selecting one of six trial moves–insertion or deletion of either FTC, ETC, or C. If an insertion move for FTC or ETC is selected then a configuration of the species is selected from a pre-sampled equilibrium Boltzmann library [1]. This configuration is then randomly rotated. In our case we generated libraries of 10,000 samples for FTC and ETC using standard Markov chain Monte Carlo. The inserted molecule is then uniformly placed in the insertion volume V_0 and velocities are assigned according the Boltzmann distribution. If a deletion move is selected, a uniformly chosen molecule of the species is removed from the simulation box (if there is at least one molecule of the species present). In either case, the trial move is conditionally accepted, with rejected moves returning the system to its original state. Following the GCMC move, the system undergoes Langevin dynamics for a certain number of time steps until the GCMC procedure is repeated. With Langevin time step $\Delta t = 0.005$ we find that 100 time steps between GCMC trial moves is sufficient to maintain equilibrated chemostats.

The relationship between the external chemical potential μ_i and the internal composition is given by

$$\mu_i = A_i^0 + \frac{1}{\beta}\log \gamma_i \frac{\langle C_i\rangle}{C_i^0}. \tag{B8}$$

With concentration $C_i = N_i/V$ and reference concentration $C_i^0 = 1/V$, the relationship between chemical potential and composition simplifies to

$$\mu_i' = \frac{1}{\beta}\log \gamma_i \langle N_i\rangle. \tag{B9}$$

The non-ideality of the solution is packaged into the activity coefficient γ_i. At low concentrations, the solution is ideal ($\gamma_i \approx 1$) because intermolecular interactions are small. As the concentration becomes larger these interactions start to dominate and the relationship between chemical potential and composition becomes non-ideal.

With our motor system we only perform GCMC moves in an outer volume, away from the motor (see Fig. 1). This is done to keep the natural dynamics of the motor unperturbed by abrupt insertions and deletions. If the volume

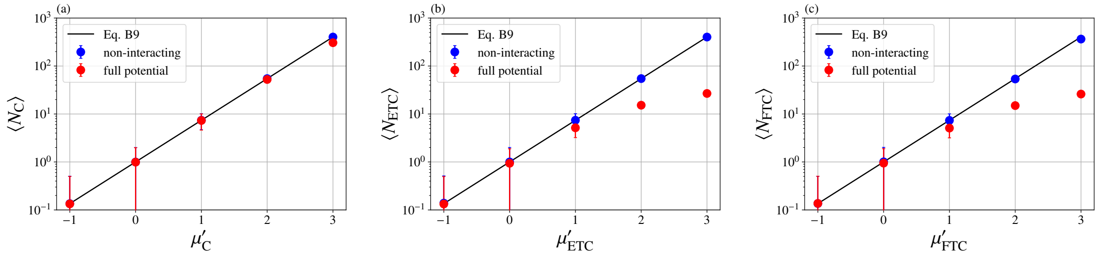

FIG. B1. **Confirming the behavior of GCMC chemostats.** Average number of species in the simulation volume of (a) C as a function of μ'_{C} with $\mu'_{\mathrm{FTC}} = \mu'_{\mathrm{ETC}} = -10$, (b) ETC as a function of μ'_{ETC} with $\mu'_{\mathrm{FTC}} = \mu'_{\mathrm{C}} = -10$, and (c) FTC as a function of μ'_{FTC} with $\mu'_{\mathrm{ETC}} = \mu'_{\mathrm{C}} = -10$. Simulation data is given for the full potential (red) as well as a non-interacting potential (blue) where intermolecular interactions are turned off. The expected relationship between μ'_i and expected average number of species for an ideal solution ($\gamma_i = 1$) given by Eq. (B9) is given for reference (black). Simulations use a time step of $\Delta t = 0.005$ with 100 Langevin dynamics time steps between attempted GCMC moves. No motors are present in the simulations and GCMC moves can occur throughout the entire volume. Error bars are given by the standard deviation over 50 independent simulations.

where GCMC moves take place is V_0, which is less than the total simulation volume accessible to the molecules V, and we assume that the diffusion of the molecules is fast relative their reaction (the diffusive Damköhler number is low) then the composition is given as

$$\langle N_i \rangle = \frac{V}{V_0}\frac{1}{\gamma_i}e^{\beta\mu'_i}. \tag{B10}$$

From Fig. C2b we see that as μ'_{FTC} is cycled between high and low values the composition of FTC reaches a new steady state very quickly, justifying this assumption. The simulations in Fig. C2 switched between $\mu'_{\mathrm{FTC}} = 0.5$ (shaded) and $\mu'_{\mathrm{FTC}} = -10$ (unshaded). The total volume $V = L^3_{\mathrm{outer}}$ with $L_{\mathrm{outer}} = 34$ with the GCMC volume $V_0 = L^3_{\mathrm{outer}} - L^3_{\mathrm{inner}}$ and $L_{\mathrm{outer}} = 30$. The inverse temperature β is 2. Assuming a dilute solution $\gamma_i \approx 1$ then from Eq. (B10) we would expect $\langle N_{\mathrm{FTC}} \rangle \approx 8.7$ for the shaded areas of Fig. C2a and $\langle N_{\mathrm{FTC}} \rangle \approx 0$ for the unshaded areas, matching the simulation data well. Thus, at these simulation parameters, the assumptions of ideal solution and low Damköhler number are justified.

As further proof that we can assume our simulations are ideal solutions and that the GCMC chemostats are working as expected, we performed tests where the entire simulation volume was available for GCMC moves and no motor was present. Those simulations were carried out with the full potential and also with non-interacting simulations where intermolecular interactions between different molecules in the simulation were not included. The ideal, non-interacting system allows for direct comparison with Eq. (B9) when $\gamma_i = 1$. As shown in Fig. B1, non-ideality begins to emerge around $\mu'_i = 1$.

Appendix C: Control Simulations

1. Chemostat Concentrations

The results of Figs. 3a and b demonstrate clear net clockwise cycling of the motor. In Fig. 3a for moderate numbers of FTC in the simulation box ($\langle N_{\mathrm{FTC}} \rangle \approx 2-8$) both Motor I and Motor II show significantly increased accuracy above the equilibrium random cycling of 50%. Both motors also show significantly increased current in this range of FTC concentration compared to an equilibrium expectation value of 0. To demonstrate that this net directional cycling is coupled to the reaction of FTC to ETC and C we carried out a series of control simulations where the external chemical potential of FTC was set to -10, low enough that no FTC is expected to appear in the simulation. The concentrations of other species were then varied to determine their effect on the motor. We varied the concentrations of ETC, C, and a variant of FTC where the free central particle was no longer free, but constrained with a FENE spring to the center of the tetrahedron. For all intents and purposes, this constrained FTC (FTC(c)) is almost identical to FTC, but cannot react to form ETC and C. The form of the constraint for FTC(c) was

$$U_{\mathrm{FENE}}(\mathbf{r}_{\mathrm{c}}) = -\frac{1}{2}k_{\mathrm{constraint}}r^2_{\mathrm{max,c}}\log\left[1-\left(\frac{|\mathbf{r}_{\mathrm{c}}|}{r_{\mathrm{max,c}}}\right)^2\right], \tag{C1}$$

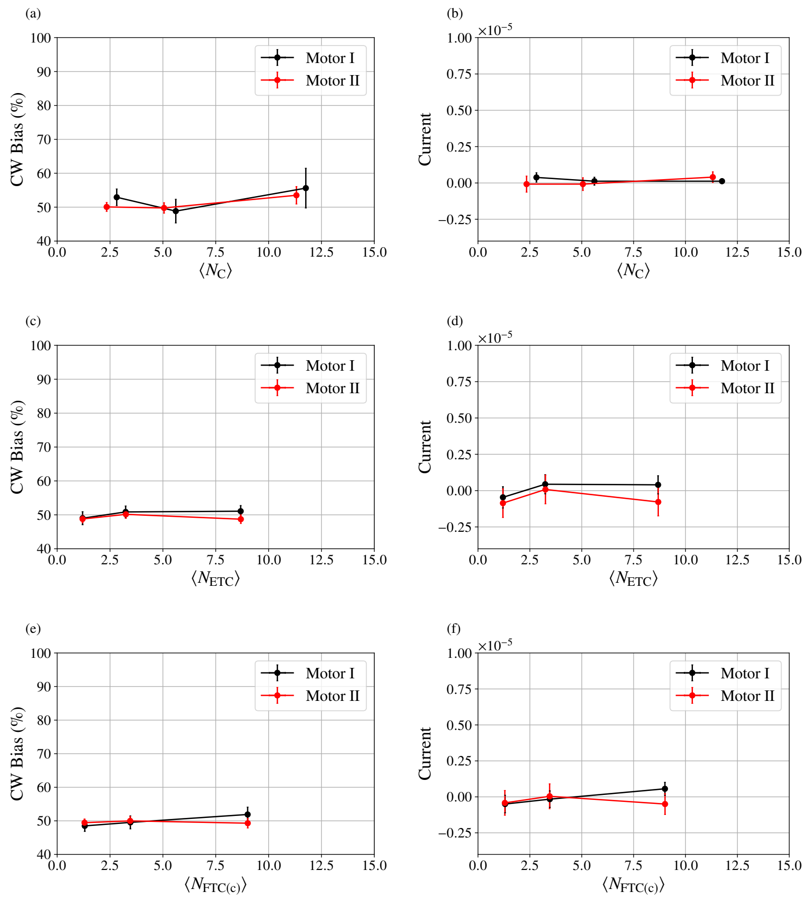

FIG. C1. **Control simulations where motor is not expected to exhibit directed motion.** The concentrations of C (a, b), ETC (c, d), and FTC(c) (e, f) are varied and clockwise bias (a, c, e, Eq. (3)) and current (b, d, f, Eq. (4)) are examined for Motor I and Motor II. Each data point represents 50 independent simulations of 1×10^6 units of time with the mean plotted and the standard error of the mean represented by error bars.

where $\mathbf{r}_c$ is the vector between the central particle and the center of mass of the four tetrahedron particles, $k_{\text{constraint}} = 120$, and the maximum extension, $r_{\text{max,c}}$ is given as the maximum distance that defines the FTC molecule as being in a reactant state, 0.25 in our case.

The results of these control simulations, presented in Fig. C1, show that for all three species ETC, C, and FTC(c) there is no deviation from an accuracy of 50% and a current of 0. In other words, these simulations are all equilibrium simulations as the decomposition of FTC and its replenishment through the external chemical potential is required to establish a nonequilibrium steady state.

2. Chemostat Timescales

To realize current, it was necessary that the chemostats behave sufficiently quickly that NESS concentrations of FTC, ETC, and C could be achieved significantly faster than the timescale for shuttling ring diffusion. We check that this criterion is satisfied by periodically switching from $\mu'_{\text{FTC}} = 0.5$ to $\mu'_{\text{FTC}} = -10$, while holding fixed $\mu'_{\text{ETC}} = \mu'_{\text{C}} = -10$. The responses to this time-periodic perturbation, plotted in Fig. C2, show that the FTC and ETC concentrations relax to their NESS values very rapidly, while the concentration of C relaxes more gradually due to its attractive interactions with the catalytic sites of the motor. Even with this slower relaxation, that central particle equilibrates much faster than the typical timescale to complete cycles. Consequently, the periodically driven motor alternates between regimes of fueled directed motion (shaded) and unfueled equilibrium diffusion (unshaded). When FTC is present the motor has a clear bias toward the CW direction and when FTC is not present the motor reverts to unbiased diffusion where it can cycle CW or CCW with equal probability, resulting in no net current.

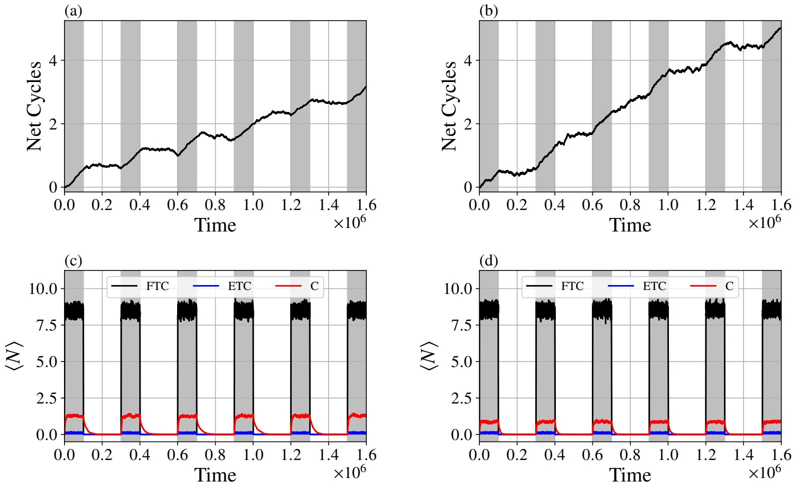

FIG. C2. **Response of motors to time varying fuel concentration.** The composition of the Motor I system (a) and Motor II system (b) are shown with periodically varying μ'_{FTC}. Average numbers of FTC, ETC, and C are given as a function of simulation time. The FTC chemical potential switches between $\mu'_{\mathrm{FTC}} = 0.5$ for 1×10^5 units of time (gray shaded regions) to -$\mu'_{\mathrm{FTC}} = -10$ for 1×10^5 for 2×10^5 units of time (unshaded regions) and then the cycle repeats. The corresponding responses of Motor I and Motor II to the periodically varying μ'_{FTC} are given in (c) and (d), respectively. Net cycles are defined as the number of clockwise cycles minus the number of counterclockwise cycles. Data shown are averages taken over 200 independent simulations.

Appendix D: Forcefield Parameters

The model consists of 11 different particle types: the two binding sites on the large ring (BIND), 22 inert sites on the large ring (INERT), six catalytic sites (2 each of CAT1, CAT2, and CAT3), 12 sites around the shuttling ring (SHUTTLE), four different types of particles on the vertices of the tetrahedra (TET1, TET2, TET3, and TET4), and the central particle (CENT). The forcefield is fully defined by the masses and radii of these particles (Table I), the pairwise interactions (Table II), and the three-body angular interactions along the backbone of the rings (Table III).

**Particle Type**	m_i	σ_i
BIND	1.0	1.0
INERT	1.0	1.0
CAT1	1.0	1.0
CAT2	1.0	1.0
CAT3	1.0	1.0
SHUTTLE	1.0	1.0
TET1	1.0	1.0
TET2	1.0	1.0
TET3	1.0	1.0
TET4	1.0	1.0
CENT	1.0	0.45

TABLE I. All particles have unit radius and unit mass except for the central particle, which has a smaller radius.

Interaction	$\epsilon_{\mathrm{R},ij}$	$\epsilon_{\mathrm{A},ij}$	Bond Type	k_{ij}	$r_{\max,ij}$
TET*-TET*	10.0	0.0	harmonic	120.0	-
INERT-INERT	1.0	0.0	FENE	30.0	1.5
INERT-BIND	1.0	0.0	FENE	30.0	1.5
BIND-CAT2	1.0	0.0	FENE	30.0	1.5
CAT2-CAT1	1.0	0.0	FENE	30.0	1.5
CAT1-CAT3	1.0	0.0	FENE	30.0	1.5
CAT3-INERT	1.0	0.0	FENE	30.0	1.5
SHUTTLE-SHUTTLE	1.0	0.0	FENE	30.0	1.5
SHUTTLE-CAT1	1.0	0.0	none	-	-
SHUTTLE-CAT2	1.0	0.0	none	-	-
SHUTTLE-CAT3	1.0	0.0	none	-	-
SHUTTLE-TET*	500000.0	0.0	none	-	-
SHUTTLE-CENT	100000.0	0.0	none	-	-
SHUTTLE-INERT	1.0	0.0	none	-	-
INERT-TET*	1.0	0.0	none	-	-
INERT-CENT	1.0	0.0	none	-	-
INERT-CAT1	1.0	0.0	none	-	-
INERT-CAT2	1.0	0.0	none	-	-
BIND-BIND	1.0	0.0	none	-	-
BIND-CAT1	1.0	0.0	none	-	-
BIND-CAT3	1.0	0.0	none	-	-
BIND-TET*	1.0	0.0	none	-	-
BIND-CENT	1.0	0.0	none	-	-
CAT1-CAT1	1.0	0.0	none	-	-
CAT2-CAT2	1.0	0.0	none	-	-
CAT2-CAT3	1.0	0.0	none	-	-
CAT3-CAT3	1.0	0.0	none	-	-
TET*-CENT	1.0	0.0	none	-	-
CENT-CENT	100000.0	0.0	none	-	-
BIND-SHUTTLE	1.0	**0.8** | **0.7**	none	-	-
CAT1-TET1	0.888468	0.7328772	none	-	-
CAT1-TET2	1.215096	0.5734710	none	-	-
CAT1-TET3	1.424995	0.2213622	none	-	-
CAT1-TET4	0.216518	0.5113107	none	-	-
CAT1-CENT	2.798031	**4.0973389** | **3.9229033**	none	-	-
CAT2-TET1	1.153983	0.4533399	none	-	-
CAT2-TET2	4.138882	1.7037981	none	-	-
CAT2-TET3	1.073067	1.5169752	none	-	-
CAT2-TET4	0.560281	0.8455581	none	-	-
CAT2-CENT	1.824090	1.456227	none	-	-
CAT3-TET1	2.973898	1.3987962	none	-	-
CAT3-TET2	1.735298	2.0515572	none	-	-
CAT3-TET3	1.808303	0.2544759	none	-	-
CAT3-TET4	3.437772	1.4306526	none	-	-
CAT3-CENT	1.019612	0.931202	none	-	-

TABLE II. Pairwise interaction parameters for both modified Lennard-Jones interactions (Eq. (10)) and bonding interactions. Vertices of the tetrahedra are linked with harmonic bonds while neighboring particles around the two rings are bonded together with FENE (finitely extensible nonlinear elastic) bonds in the order: BIND, CAT2, CAT1, CAT3, 11 INERT, BIND, CAT2, CAT1, CAT3, 11 INERT. For harmonic bonds k_{ij} is that of Eq. (11) while for FENE it is the $k_{\mathrm{F},ij}$ of Eq. (12). The two bold entries report the value for Motor I | the value for Motor II. An asterisk in the particle name, e.g. TET*, indicates that the value is the same for TET1, TET2, TET3, and TET4.

Interaction	$k_{\mathrm{A},ijk}$	$\theta_{0,ijk}$
SHUTTLE-SHUTTLE-SHUTTLE	3.0	$\pi\left(1-\frac{2}{N_{\mathrm{shuttle}}}\right)$
BIND-CAT2-CAT1	10.0	$\pi\left(1-\frac{2}{N_{\mathrm{large}}}\right)$
CAT2-CAT1-CAT3	10.0	$\pi\left(1-\frac{2}{N_{\mathrm{large}}}\right)$
CAT1-CAT3-INERT	10.0	$\pi\left(1-\frac{2}{N_{\mathrm{large}}}\right)$
CAT3-INERT-INERT	10.0	$\pi\left(1-\frac{2}{N_{\mathrm{large}}}\right)$
INERT-INERT-INERT	10.0	$\pi\left(1-\frac{2}{N_{\mathrm{large}}}\right)$
INERT-INERT-BIND	10.0	$\pi\left(1-\frac{2}{N_{\mathrm{large}}}\right)$
INERT-BIND-CAT2	10.0	$\pi\left(1-\frac{2}{N_{\mathrm{large}}}\right)$

TABLE III. Angular interaction parameters for Eq. (13). Note that the angular interactions are symmetric about the center particle such that $k_{\mathrm{A},ijk}$ would be equivalent to $k_{\mathrm{A},kji}$.

[1] S. Chempath, L. A. Clark, and R. Q. Snurr, The Journal of Chemical Physics **118**, 7635 (2003).
