# Supplementary figures and images for: Simulating a chemically fueled molecular motor with nonequilibrium molecular dynamics
Source: Nat Commun. 2022 Apr 22;13:2204. doi: 10.1038/s41467-022-29393-3 (PMC9033874; doi:10.1038/s41467-022-29393-3)

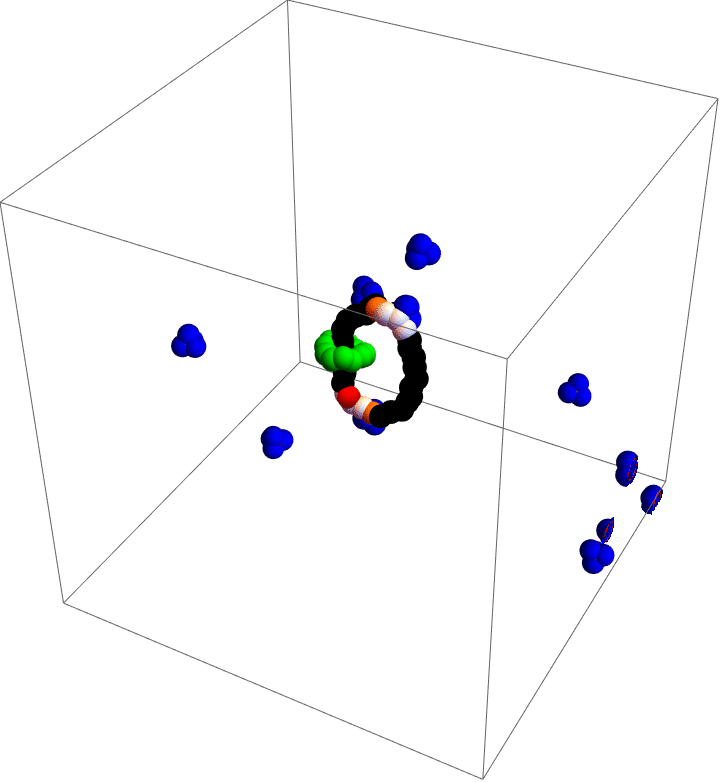

Supplement: Supplementary file 3 — Supplementary Movie 1 [file 41467_2022_29393_MOESM3_ESM.gif]

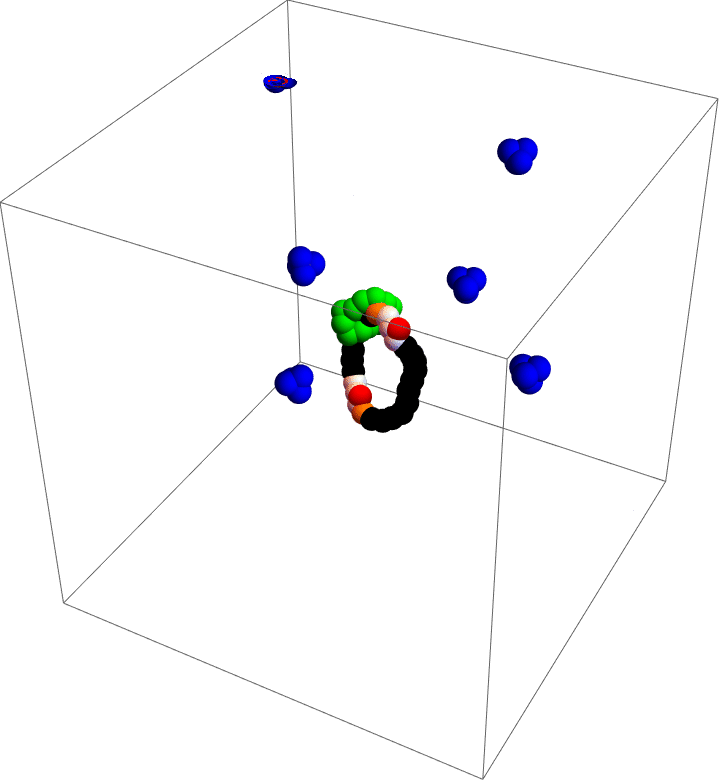

Supplement: Supplementary file 4 — Supplementary Movie 2 [file 41467_2022_29393_MOESM4_ESM.gif]

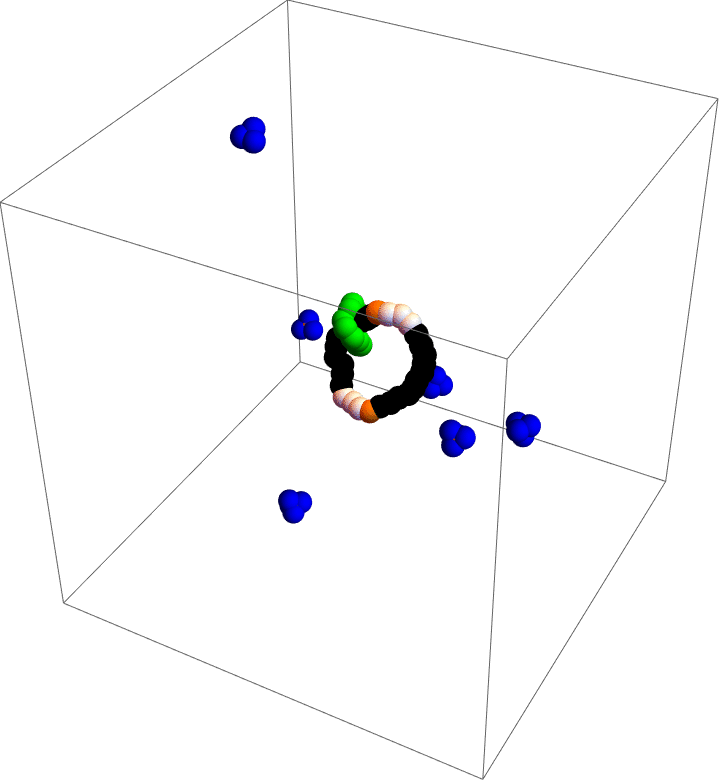

Supplement: Supplementary file 5 — Supplementary Movie 3 [file 41467_2022_29393_MOESM5_ESM.gif]
